# Overexpression of *SlOFP20* affects floral organ and pollen development

**DOI:** 10.1038/s41438-019-0207-6

**Published:** 2019-11-15

**Authors:** Shengen Zhou, Zongli Hu, Fenfen Li, Shibing Tian, Zhiguo Zhu, Anzhou Li, Guoping Chen

**Affiliations:** 1 Laboratory of Molecular Biology of Tomato, Bioengineering College, Chongqing University, Chongqing, People’s Republic of China; 2 Institute of Vegetable Research, Chongqing Academy of Agricultural Sciences, Chongqing, People’s Republic of China

**Keywords:** Plant molecular biology, Pollen

## Abstract

The *OVATE* gene was initially identified in tomato and serves as a key regulator of fruit shape. There are 31 OFP members in the tomato genome. However, their roles in tomato growth and reproductive development are largely unknown. Here, we cloned the *OFP* transcription factor *SlOFP20*. Tomato plants overexpressing *SlOFP20* displayed several phenotypic defects, including an altered floral architecture and fruit shape and reduced male fertility. *SlOFP20* overexpression altered the expression levels of some brassinosteroid (BR)-associated genes, implying that SlOFP20 may play a negative role in the BR response, similar to its ortholog OsOFP19 in rice. Moreover, the transcript accumulation of gibberellin (GA)-related genes was significantly affected in the transgenic lines. SlOFP20 may play an important role in the crosstalk between BR and GA. The pollen germination assay suggested that the pollen germination rate of *SlOFP20*-OE plants was distinctly lower than that of WT plants. In addition, the tomato pollen-associated genes *SlCRK1*, *SlPMEI*, *LePRK3*, *SlPRALF*, and *LAT52* were all suppressed in the transgenic lines. Our data imply that *SlOFP20* may affect floral organ and pollen development by modulating BR and GA signaling in tomato.

## Introduction

The reproductive development of most higher plants includes floral meristem determination, floral bud emergence, and fruit development and ripening, all of which result in seed formation and dispersal to guarantee offspring survival^1^. Numerous studies indicate that several gene families extensively participate in the transcriptional modulation of reproductive and developmental processes. For example, the microRNA156-targeted SPL/SBP box transcription factors control the processes of ovary and fruit development in tomato^2^. Silencing of *SlDELLA*, a GRAS transcription factor, induces facultative parthenocarpy in tomato fruits. A gain-of-function mutation in the MADS-box gene *Sl-AGL11* exerts a great impact on the organization of flowers and fruit, especially on the transition of the sepals into a carpel-like fleshy organ, and on increases in sugar content and fruit softness^3^. The OVATE family proteins (OFPs) are plant-specific transcription factors. The *OVATE* gene was originally characterized as a key quantitative trait locus that contributes to the conversion of tomato fruit from round to pear shaped^4^. Amino acid sequence analysis shows that this gene encodes a hydrophilic protein including a putative bipartite nuclear localization signal and a C-terminal domain of ~70 amino acids referred to as the OVATE domain. Unlike other known transcription factor families, the OVATE proteins are a novel class of functional proteins^5^. OFPs have mainly been functionally characterized in *Arabidopsis*^6–11^ and rice^12–15^ and have been demonstrated to control diverse aspects of plant growth and development. Overexpression of *AtOFP1* decreases the length of all aboveground organs, such as the hypocotyl, rosette leaf, floral organs and siliques, and chromatin immunoprecipitation analysis demonstrated that AtOFP1 directly regulates *AtGA20ox1*, encoding the key enzyme in GA biosynthesis^7^. Additionally, AtOFP1 may take part in DNA repair^8^. Moreover, plants overexpressing *AtOFP2*, *4* and *7* generate similar phenotypes to *AtOFP1*-overexpressing plants, such as kidney-shaped cotyledons and round and curled leaves^7,10^. Overexpression of *OsOFP2* results in decreased plant height and an altered leaf morphology and seed shape in rice^12^. Overexpression of *OsOFP1* in rice causes multiple phenotypes, including increased leaf angles, decreased plant height, and altered grain shape^14^. The interaction of MaOFP1 and MuMADS1 in banana plays an antagonistic role in ethylene-induced postharvest fruit ripening^16^. According to the most recent study, MuMADS1 and MaOFP1 control fruit quality in a tomato *ovate* mutant^17^. These results strongly support the notion that OFPs act as important regulatory factors in numerous processes in plant growth and development.

Studies on the mechanism of action of OFPs have shown that they function via interacting with different kinds of transcription factors, such as KNOX and BELL classes^6,13,18^. The interaction networks between OFPs and TALE proteins have an important effect on plant developmental processes. The interaction of AtOFP1 and BLH3 has been shown to regulate the timing of conversion from the vegetative to reproductive stage in *Arabidopsis*^11^. AtOFP4 has been proposed to interact with KNAT7 (Knotted1-Like Homeodomain Protein 7) to control the establishment of secondary cell walls by increasing the transcriptional repression activity of KNAT7^10^. The interaction of AtOFP5 with KNAT3 and BLH1 inhibits the activity of BELL–KNOX TALE complexes to assure normal embryo sac development in *Arabidopsis*^19^. In *Arabidopsis*, the cell wall defect of the *knat7* mutant can be partially restored by ectopic expression of *GhKNL1*, a homeodomain protein from cotton (*Gossypium hirsutum*); moreover, GhKNL1 can interact with GhOFP4, AtOFP1, and AtOFP4^20^.

Hormone pathways extensively participate in the extraordinary plasticity of plant ontogeny. There are several classes of phytohormones, including auxins, brassinosteroids, and gibberellins, that play essential roles in the regulation of growth in general and of cell elongation in particular^21^. Previous reports suggest that OFPs affect plant developmental processes by modulating the brassinosteroid and gibberellin signaling pathways^7,13–15^. AtOFP1 controls cell elongation in part by regulating the mRNA accumulation of the gibberellin biosynthesis gene *AtGA20ox1*^7^. OsOFP19, OSH1 and DLT form a complex that regulates the complicated balance between plant growth and development and brassinosteroid signaling^15^. Increased brassinosteroid signaling can induce the expression of *OsOFP1* by OsBZR1 and promote protein stability by repressing OsGSK2, resulting in the activation of OsOFP1, which then bonds with DLT factors and regulates downstream genes such as gibberellin metabolism genes to control plant morphology and grain shape in rice^14^. OsOFP8 acts as a positive regulator in the brassinosteroid signaling pathway by interacting with OsGKS2, which plays a negative role in the brassinosteroid signaling pathway^13^.

In view of the outstanding nutritive and commercial value of tomato (*Solanum lycopersicum*), it has been regarded as one of the most important vegetable crops. It is also a model organism for studying fleshy fruit development and ripening^22^, compound leaf development, and floral system and plant architecture^23^. Genome-wide analysis of OFPs in tomato has been carried out, and there are 31 SlOFPs in the tomato genome^24^. Herein, we present the functional characterization of *SlOFP20* (accession number: Solyc10g076180), a classic OFP family gene homologous to *AtOFP1* and *OsOFP19* in *Arabidopsis* and rice, respectively. It has been reported that *SlOFP20* is a suppressor of *ovate* in the modulation of fruit shape^25^. To investigate the role of *SlOFP20* related to the development of vegetative and reproductive growth in tomato, the *SlOFP20* gene was cloned and overexpressed in wild-type tomato, leading to pleiotropic phenotypes. In this study, we sought to reveal the impacts of *SlOFP20* on reproductive development, including floral architecture and pollen development. Morphological, statistical, and molecular evidence is reported here to clarify the potential reasons for these phenotypes.

## Materials and methods

### Plant materials and growth conditions

*Solanum lycopersicum* Mill. cv. Ailsa Craig tomato plants were used as the wild-type (WT) in our research. WT and transgenic tomato plants were grown in a greenhouse under standard greenhouse conditions (16-h-day/8-h-night cycle, 25 °C/18 °C day/night temperature). To determine the organ-specific expression pattern of *SlOFP20*, roots, stems, leaves, sepals, flowers, and fruits of different stages were sampled from WT tomato plants according to our previous report^26^. The four-whorl floral organs (sepal, petal, stamen, and carpel) were also harvested. For 24-epibrassinolide (EBR) treatment, 10 μM EBR and water (control) were sprayed on five-leaf-stage wild-type tomato plants. In addition, the third leaves from treated and untreated plants were harvested after 0, 1, 2, 4, 8, 12, and 24 h. All samples used in this study were immediately frozen with liquid nitrogen and kept at −80 °C.

### Sequence analysis and phylogenetic tree construction

The protein sequence alignment of SlOFP20 and other OFP proteins was generated by using the DNAMAN 5.2.2 programs. The conserved OVATE domains were identified by using Scan Prosite (http://prosite.expasy.org/scanprosite/) to reveal the phylogenetic relationships of SlOFP20 with 17 OFP family proteins from *Arabidopsis* and rice. The maximum likelihood (ML) method was applied to construct a dendrogram with MEGA 6.06 software. The accuracy of this tree was ensured by the bootstrap test replicated 1000 times. The GenBank accession numbers of the proteins included in the tree were as follows: AtOFP1 (NP_195804), AtOFP2 (NP_180599), AtOFP4 (NP_172174), AtOFP5 (NP_193618), AtOFP6 (NP_680125), AtOFP7 (NP_179440), AtOFP8 (NP_197466), AtOFP13 (NM_196102), AtOFP15 (XM_565833), AtOFP16 (NP_180770), AtOFP18 (NP_566967), OsOFP1 (XP_015643684), and OsOFP19 (XP_015638848). The Tomato Solanaceae Genomics Network (SGN) unigene accession numbers were as follows: SlOFP5 (Solyc02g072030), SlOFP14 (Solyc06g082460), SlOFP15 (Solyc07g055240), SlOFP17 (Solyc09g018200), and SlOFP20 (Solyc10g076180).

### Vector construction and tomato transformation

For the overexpression of *SlOFP20* in WT tomato, the full-length sequence was amplified by high-fidelity PCR (Prime START mix DNA polymerase, Takara) with the *SlOFP20*-F and *SlOFP20*-R primers (Supplementary Table S1), which were tailed with *Xba*I and *Sac*I restriction sites, respectively, at their 5′ ends. A DNA-Tailing kit (Takara) was applied to tail the obtained PCR products, which were then linked with the pMD18-T vector (Takara). The correct pMD18-T-SlOFP20 plasmid was used as the template and was amplified with the primers *SlOFP20*-F and *SlOFP20*-R. Then, the amplified products were inserted into the pBI121 vector. The resulting *SlOFP20*-OE vector was introduced into *Agrobacterium* LBA4404. Plant transformation was conducted following our previously published protocols^27^. Transgenic lines were screened on kanamycin medium and verified by genomic PCR using the *NPTII*-F and *NPTII*-R primers (Supplementary Table S1). The positive *SlOFP20*-OE transgenic lines were retained and used for further studies.

### Gene expression analysis

In this study, RNAiso Plus (Takara) was used to extract total RNA. One microgram of total RNA was reverse-transcribed (M-MLV Reverse Transcriptase Kit, Promega). Transcript levels were evaluated by real-time quantitative PCR according to a method published previously^28^. S*lCAC* (Solyc08g006960) was used as a reference gene^29^. The primer sequences are shown in Supplementary Table S1.

### Transactivation activity and yeast two-hybrid assay for SlOFP20

Transactivation activity and yeast two-hybrid assays were performed according to our previous report^26^.

### Anatomic characterization and scanning electron microscopy

For anatomic characterization, WT and OE3 flowers at anthesis were fixed with 70% ethanol/acetic acid/formaldehyde (18:1:1, v/v/v). Paraffin sections were prepared according to a previous report^30^. Transverse sections of the middle part of the flowers were observed with a microscope (OLYMPUS IX71) and photographed.

For SEM (scanning electron microscopy) analysis, fully open flowers from the WT and OE3 transgenic lines were collected and fixed with 2% glutaraldehyde. The samples were dehydrated in a gradient ethanol-water series. After vacuum drying, the stamens were separated and coated with gold for SEM observation and photography.

### Pollen germination and viability assays

Pollen germination was performed as described previously^31^. Briefly, WT and OE3 transgenic plant anthers at the anthesis stage were collected and transferred to germination solution. Released pollen was then germinated by incubation in the dark at 25 °C for 3 h and were defined as germinated when the pollen tube was at least as long as the diameter of the pollen grain. Pollen germination was observed, and images were taken. A pollen viability assay was conducted as described previously^32^. Pollen grains of WT and OE3 plants at the anthesis stage were collected and soaked in a 0.1% 2,3,5-triphenyl-2 h-tetrazolium chloride (TTC) solution for 15 min to assess their activity. The experiments were repeated three times.

### Cross assay

A cross assay was performed according to our previous report^33^. In brief, unopened flower buds from OE3 transgenic lines were emasculated. Mature pollen from the WT was transferred by brushing the WT anthers onto the stigmas of the OE3 transgenic lines.

### Seed germination assay

Seeds from WT and *SlOFP20*-OE transgenic tomato lines (T2) were used for germination assays. After surface sterilization, seeds (~30 seeds for each replicate) were sown onto MS medium and then germinated in the dark at 25 °C for 10 days. Radicle emergence >1 mm was regarded as seed germination. Seed germination rates were recorded daily. The experiments were repeated three times.

## Results

### SlOFP20 is a typical OVATE family protein

**Fig. 1:**
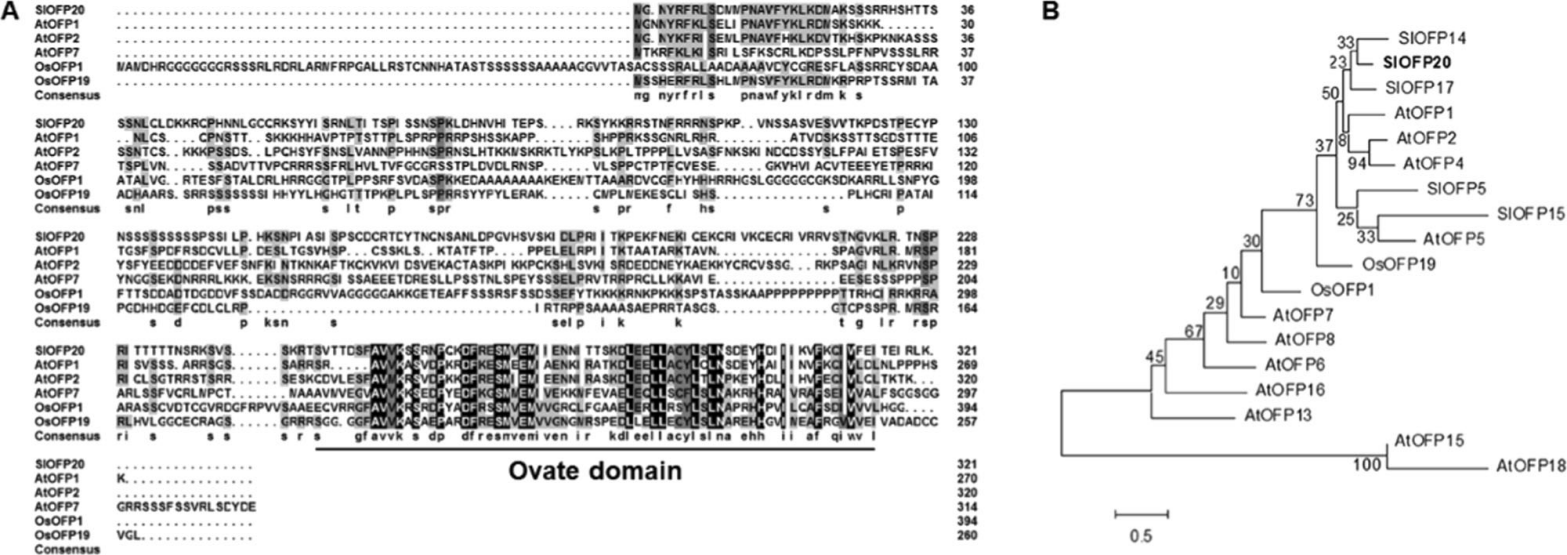
Amino acid sequence comparison and phylogenetic tree of SlOFP20 and other OVATE family proteins. **a** Multiple sequence alignment of the SlOFP20, AtOFP1, AtOFP2, AtOFP7, OsOFP1, and OsOFP19 proteins. The black and gray shading show amino acids that are identical or similar, respectively. The conserved OVATE domain in the C-terminus is underlined. **b** The phylogenetic tree of SlOFP20 and other OVATE family proteins was constructed to show the relationships between *Arabidopsis* and rice OVATE family proteins and OVATE family proteins from tomato

OVATE family proteins govern various developmental processes. To study the potential roles of OFP genes in tomato, we isolated an OFP gene (*SlOFP20*) from WT tomato on the basis of the sequence available in GenBank (accession no. XM_004248997). The *SlOFP20* gene is intronless and has an open reading frame (ORF) of 966 nucleotides, which encodes a protein of 321 amino acid residues. Multiple sequence alignment analysis of SlOFP20 and other well-known OVATE family proteins from *Arabidopsis* and rice indicated that the SlOFP20 protein possesses a typical OVATE domain in the C-terminus (Fig. 1a). Phylogenetic analysis was carried out to study the relationship between tomato SlOFP20 and members of the *Arabidopsis* and rice OVATE family proteins (Fig. 1b), revealing that this tomato protein can be classified into a distinct clade that includes AtOFP1 and OsOFP19, its putative orthologues from *Arabidopsis* and rice, respectively.

A yeast two-hybrid system was applied to study the transcriptional activity of SlOFP20. A GAL4 DNA-binding domain SlOFP20 fusion protein was expressed in Y2H yeast cells to evaluate their capacity to initiate transcription from the GAL4 sequence. SlOFP20 could not promote yeast growth in the absence of histidine and adenine (Fig. S1), indicating that SlOFP20 does not exhibit transactivation activity.

### Expression patterns of *SlOFP20*

**Fig. 2:**
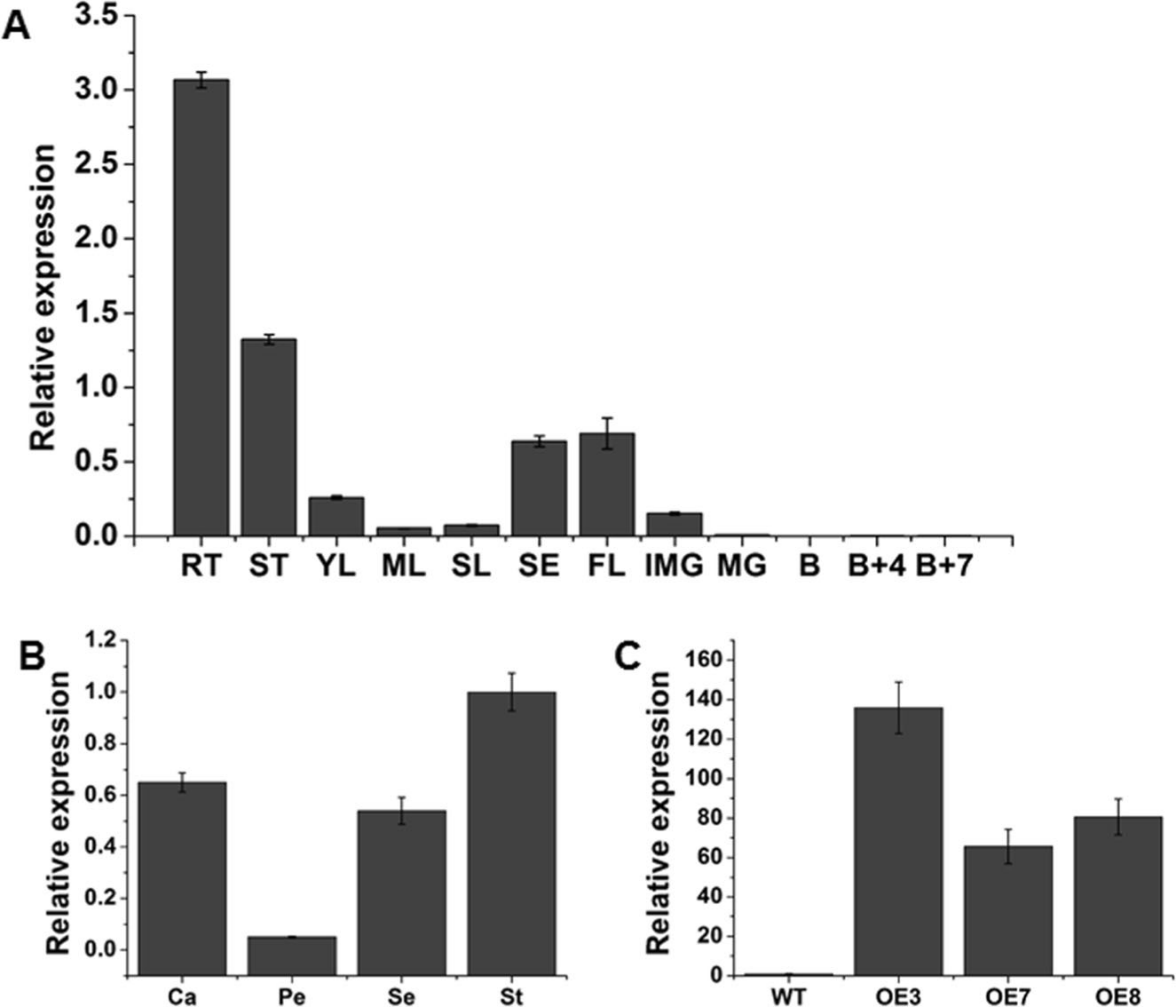
Expression profile analysis of *SlOFP20* in WT tomato plants by quantitative real-time PCR. **a** Expression levels of *SlOFP20* genes in different tissues of WT tomato. Rt, root; St, stem; Yl, young leaf; Ml, mature leaf; Sl, senescent leaf; S, sepal; Fl, flower; IMG, immature green fruit; MG, mature fruit; **b** breaker fruit; B+4, 4 days after the breaker stage; B+7, 7 days after breaker stage. **b** Relative expression of *SlOFP20* in the four-whorl floral organs of WT. Se, sepal; Pe, petal; St, stamen; Ca, carpel. **c** The expression levels of *SlOFP20* in the WT and transgenic lines. Each value represents the mean ± SE of three replicates

To predict the potential function of *SlOFP20* underlying tomato growth and development, quantitative reverse transcription-PCR (RT-qPCR) was applied to examine its expression patterns in various tomato organs. As shown in Fig. 2a, the results suggested that *SlOFP20* showed the highest transcript accumulation in the roots, followed by the stems and flowers, while relatively low transcript levels were present in the leaves and IMG fruits. *SlOFP20* mRNA was not detected in MG, B, B+4, and B+7 fruits. In addition, the expression level of *SlOFP20* in the four-whorl flower organs in WT tomato was analyzed, indicating that *SlOFP20* was mainly present in the sepals, stamens, and carpels (Fig. 2b). These results suggested that *SlOFP20* showed tissue-specific expression in tomato and may be involved in the development of roots, stems, and flowers.

### Overexpression of *SlOFP20* alters tomato flower and fruit morphology

**Fig. 3:**
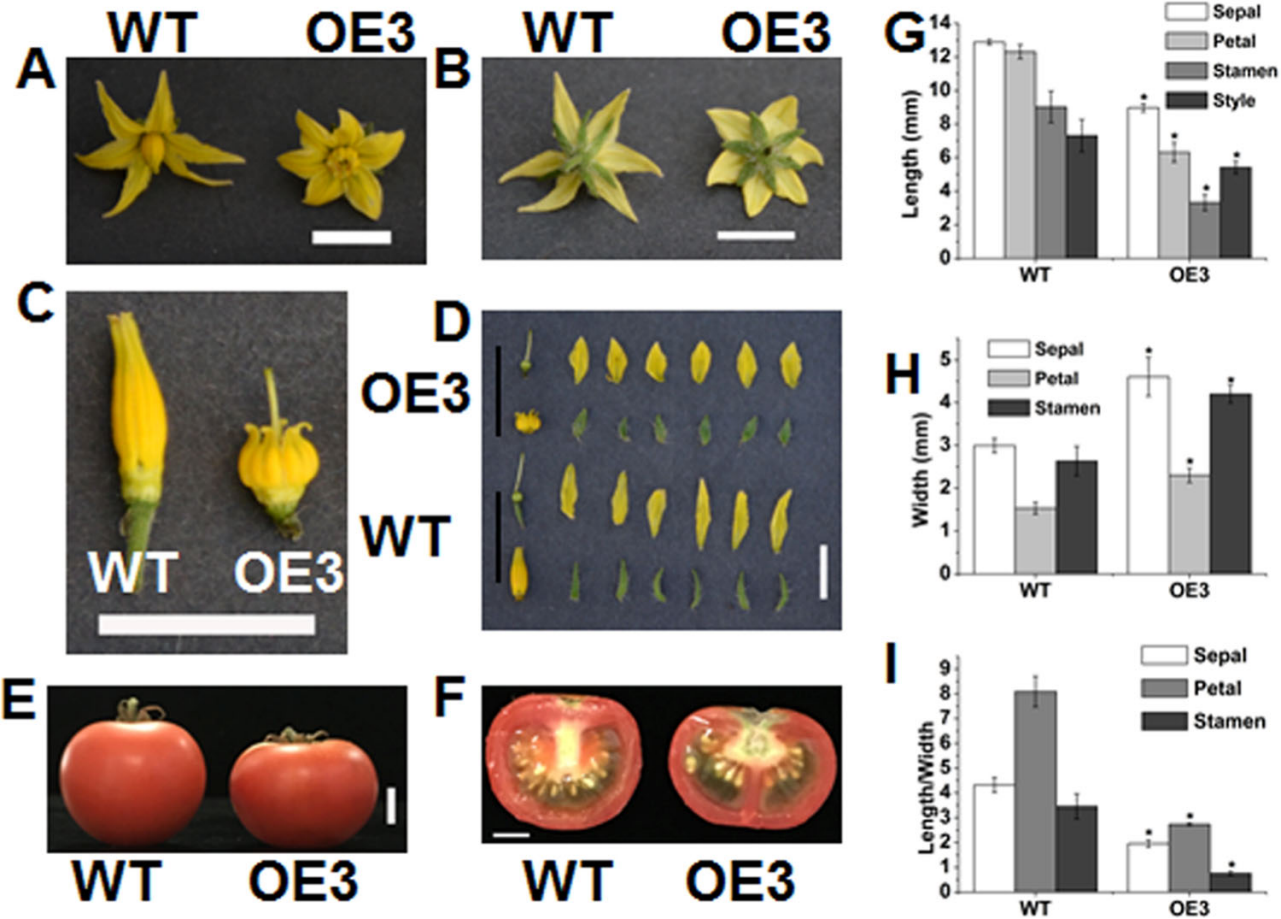
Morphological changes displayed by *SlOFP20*-OE transgenic lines. **a**, **b** The overexpression of *SlOFP20* resulted in altered flower size. Bar = 1 cm. **c** Stigma exsertion phenotype of an *SlOFP20* overexpression line. Bar = 1 cm. **d** The four-whorl floral organs of an *SlOFP20* overexpression line (upper row) and WT (lower row). Bar = 1 cm. **e**, **f** The fruit shape of the WT and *SlOFP20* overexpression lines. Bar = 1 cm. **e**, **f** Characteristics of four-whorl floral organs from fully open WT and *SlOFP20*-OE flowers. **g**, **h** show the maximum length, maximum width, and shape index of the WT and transgenic lines, respectively. Each value represents the mean ± SE of three replicates (*n* = 9). * indicates a significant difference (*P* < 0.05) between the WT and transgenic lines

Previous studies on OFP members in *Arabidopsis* revealed that single or multiple knockout mutants of OFP members do not display morphological defects^9,12^. In contrast, overexpression of some OFP members generates obvious morphological alterations, indicating that these family members have redundant functions. There are 31 OFP family members in tomato, and *SlOFP20* is closely related to *SlOFP14*. In addition, the *Atofp1-1* mutant, in which the putative orthologous gene of *SlOFP20* in *Arabidopsis* is mutated, does not show evident morphological changes. Downregulation of *SlOFP20* in wild-type tomato does not impact fruit shape^25^. Thus, we inferred that *SlOFP20* may exhibit functional redundancy with other *OFP* members in tomato. For this reason, we overexpressed *SlOFP20* in wild-type tomato to investigate its possible functions in tomato growth and development. The full-length *SlOFP20* gene fragment was cloned into a plant overexpression vector (pBI121), which was then transferred to WT tomato. Nine independent transgenic lines were obtained. The mRNA accumulation of *SlOFP20* was upregulated in the leaves of all transgenic lines. The 3*5S:SlOFP20* plants exhibited numerous morphological defects related to vegetative and reproductive organs, indicating a prominent role of *SlOFP20* in a wide range of tomato growth and developmental phases. One of the most distinct alterations was plant sterility, observed in the strong overexpression transgenic lines. Thus, we selected the mild overexpression transgenic lines OE3, OE7, and OE8, which produced seeds, for further investigation. The representative overexpression efficiency of the T1 generation of OE3, OE7, and OE8 transgenic lines was evaluated by RT-qPCR (Fig. 2c). We also identified strong overexpression plants in the T1 generation transgenic lines, which showed a number of abnormal phenotypes, including plant growth retardation, exserted stigmas, and an altered vegetative and floral architecture. In this study, we focused on these phenotypes associated with reproductive development. Compared to WT, the flowers of OE plants were shorter, but the floral organs, including the sepals, petals, and stamens, were wider (Fig. 3a–c). Therefore, the length and width of the sepals, petals, and stamens in WT and OE3 plants were measured (Fig. 3g, h). The results showed that the lengths of the sepals, petals, and stamens were significantly reduced in OE3 plants, while the widths were greater than in WT. The shape indexes (length/width) of the sepals, petals and stamens was also calculated, which showed a remarkable reduction in OE3 plants (Fig. 3i). As shown in Fig. 3c, the flowers of OE line plants showing strong overexpression exhibited exserted stigmas, which prevented pollination and resulted in sterility; thus, we could not observe a phenotype related to tomato fruit. However, the mild overexpression plants in the T1 generation transgenic lines could produce fruits, and the changes in fruit shape resembled those in the flowers (Fig. 3e, f).

**Fig. 4:**
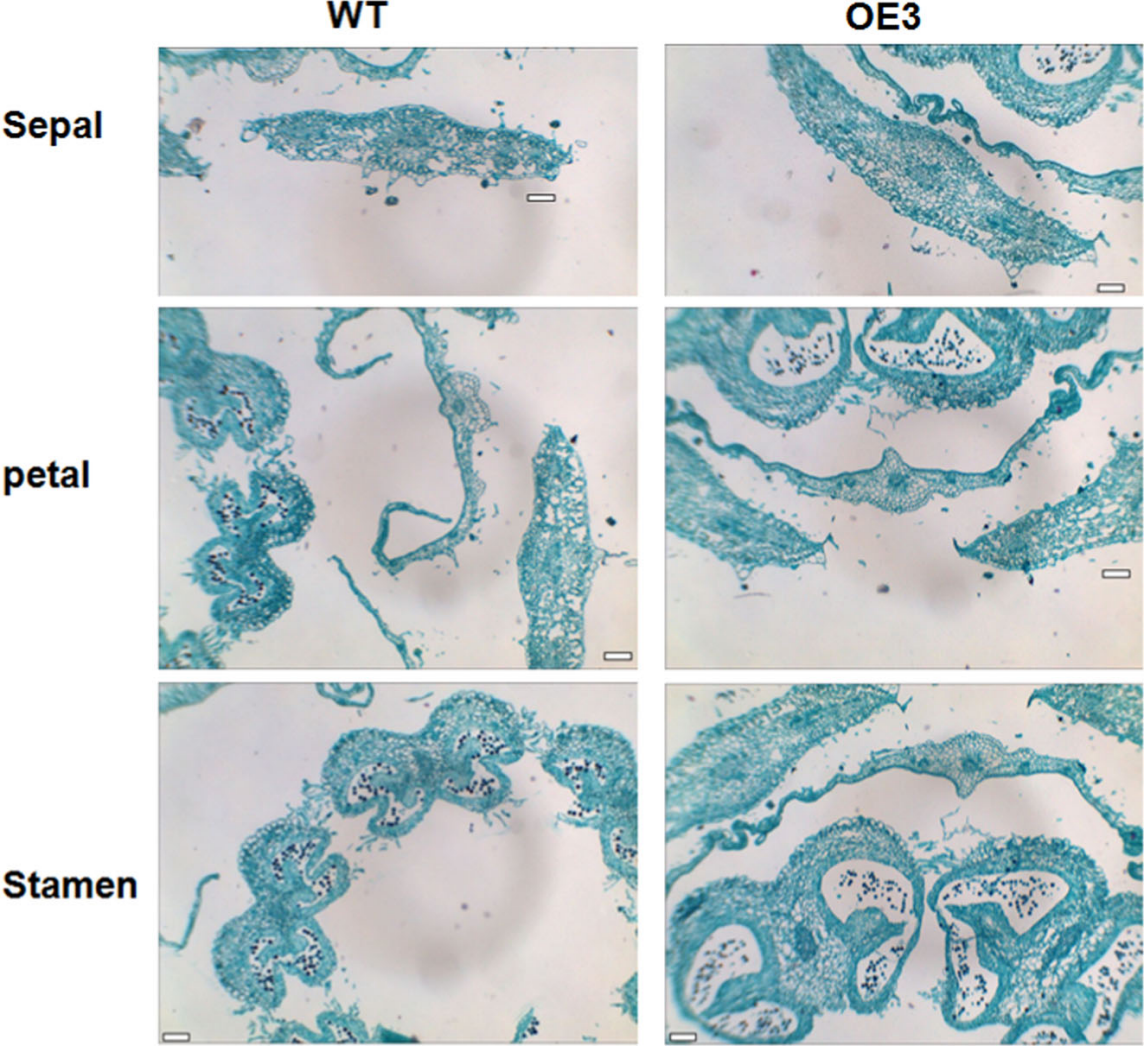
Anatomical analysis of wild-type and transgenic lines. Transverse sections of flowers from WT (left) and *SlOFP20*-OE plants (right). Bars = 100 μm

To determine the cytological difference between WT and *SlOFP20*-OE flowers, anatomical analysis of the flowers at anthesis was conducted. Compared to WT, the transverse sections of OE3 sepals, petals, and stamens were much thicker due to an increase in the cell layer number and a larger cell size (Fig. 4).

### Overexpression of *SlOFP20* in tomato affects BR- and GA-related genes

**Fig. 5:**
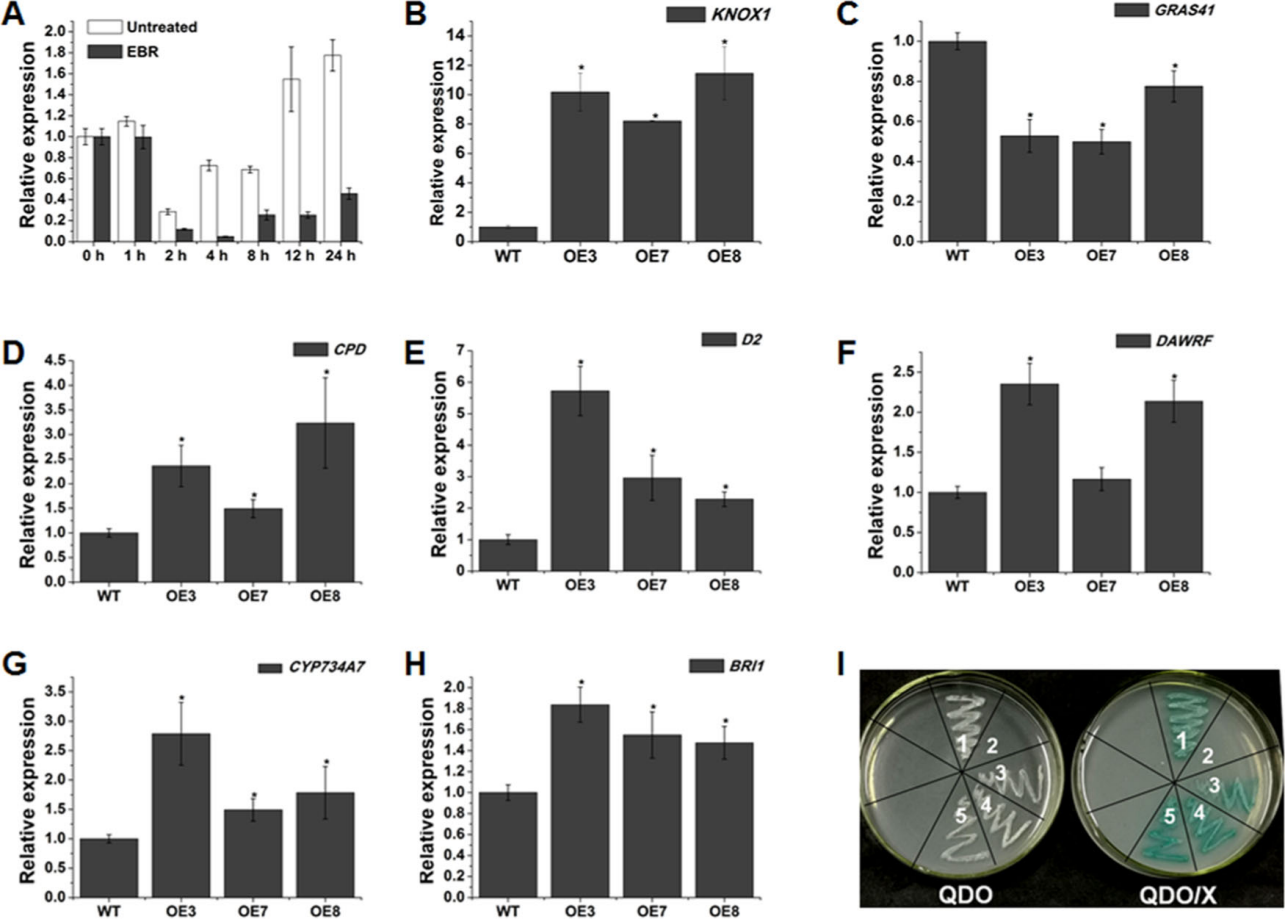
Overexpression of *SlOFP20* affects BR-related genes. **a** The expression levels of *SlOFP20* at different time points under 10 μΜ EBR treatment. **b**–**h** Comparison of BR-related gene expression between WT and overexpression lines. Each value represents the mean ± SE of three replicates. * indicates a significant difference (*P* < 0.05) between the wild-type and transgenic lines. **i** Yeast two-hybrid assay for the SlOFP20 & SlKNOX1, SlOFP20& SlGRAS41 and SlKNOX1 & SlGRAS41 proteins. QDO, SD medium without Trp, Leu, His, and Ade; QDO/X, QDO medium with X-a-Gal. 1. pGBKT7-53 & pGADT7-T (positive control); 2. pGBKT7-Lam & pGADT7-T (negative control); 3. pGBKT7-SlOFP20 & pGADT7-SlKNOX1; 4. pGBKT7-SlOFP20 & pGADT7-SlGRAS41; 5. pGBKT7-SlKNOX1& pGADT7-SlGRAS41; Empty bait vector, empty prey vector, and autoactivation assay with no growth of yeast

BR and GA are two principal phytohormones that function redundantly in promoting plant growth. The most visible phenotype of BR- and GA-deficient plants is dwarfed growth. Here, the phenotypes caused by overexpression of *SlOFP20* in tomato strongly resembled those of plants lacking BR and GA. Overexpression of *AtOFP1* in *Arabidopsis* results in reduced cell elongation, which has been partially attributed to the suppression of gibberellin biosynthesis^7^. Moreover, a recent study showed that OsOFP19 negatively regulates the brassinosteroid (BR) response in rice^15^. EBR treatment continuously inhibited the expression of *SlOFP20* (Fig. 5a). Therefore, we speculated that overexpression of *SlOFP20* inhibited plant growth by controlling the BR and GA pathways. To verify this speculation, the relative expression levels of some BR- and GA-related genes were detected by RT-qPCR. In rice, OsOFP19 increases the transcriptional activity of OSH1 but suppresses DLT^15^. The homologous genes of *OSH1* and *DLT* in tomato are *KONX1* and *GRAS41*, respectively. Similarly, the transcript level of *KNOX1* in *SlOFP20*-OE transgenic lines was sharply increased (Fig. 5b), whereas the expression of *GRAS41* was significantly inhibited compared to that in WT plants (Fig. 5c).

The expression profile of *KNOX1* obtained from the Tomato eFP Browser (http://bar.utoronto.ca/efp_tomato/cgi-bin/efpWeb.cgi) showed that KNOX1 was mainly expressed in roots and flowers (Fig. S2). The *GRAS41* transcript also primarily occurred in roots and flowers^34^. *SlOFP20*, *KNOX1*, and *GRAS41* showed similar expression patterns in tomato, implying that they may function together to regulate plant growth and development. To confirm this possibility, a yeast two-hybrid assay was used to examine their interactions. The results suggested that SlOFP20 could interact with KNOX1 and GRAS41, and KNOX1 and GRAS41 showed a definite interaction (Fig. 5i). In addition, we assessed the mRNA abundance of the BR biosynthesis genes *CPD*, *D2* (an ortholog gene of rice *OsD2*), and *DWARF* in *SlOFP20*-OE transgenic lines, and the results indicated that the levels of these BR biosynthesis genes were remarkably increased in *SlOFP20*-OE plants (Fig. 5d–f). Moreover, the BR catabolism gene *CYP734A7* was increased in *SlOFP20*-OE plants (Fig. 5g). The upregulation of BR biosynthesis genes may be due to feedback regulation, and the BR receptor BRI1 is essential for the homeostasis of endogenous BR contents^35^. Thus, the expression level of *BRI1* was measured and was shown to be significantly increased in *SlOFP20*-OE transgenic lines (Fig. 5h). Similar results were found in *OsOFP19*-OE transgenic rice plants^15^. Based on these results, we inferred that the mechanism whereby SlOFP20 regulates BR signaling may closely resemble that of its orthologue OsOFP19 from rice.

**Fig. 6:**
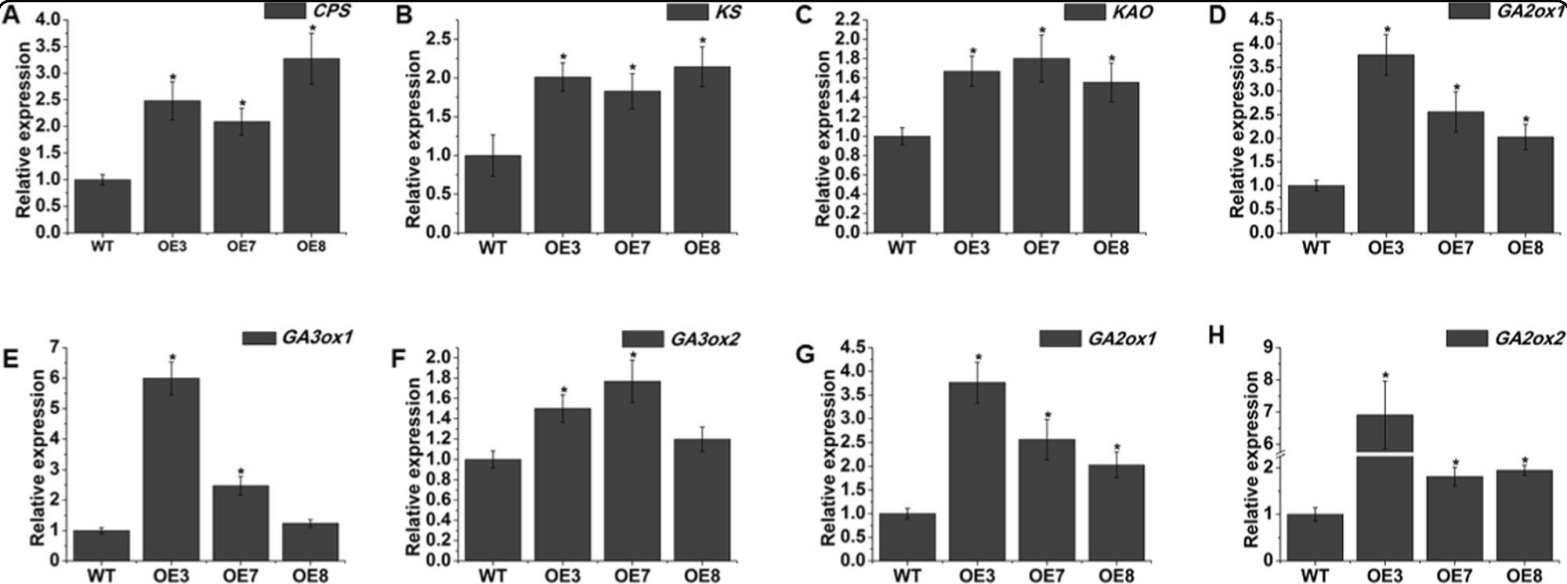
Overexpression of *SlOFP20* affects GA-related genes. Comparison of GA-related gene expression between WT and overexpression lines (**a**–**g**). Each value represents the mean ± SE of three replicates. * indicates a significant difference (*P* < 0.05) between the wild-type and transgenic lines

On the other hand, AtOFP1 reduces the mRNA accumulation of the GA biosynthesis gene *AtGA20ox1* via binding to KNAT1, corresponding to KNOX1 in tomato^6,7^. Therefore, we also evaluated the transcript accumulation of GA biosynthesis genes in wild-type and *SlOFP20*-OE plants. Three genes involved in the early steps of GA biosynthesis, *CPS*, *KS*, and *KAO*, were markedly increased in *SlOFP20*-OE transgenic plants (Fig. 6a–c). GA20oxs are also major GA biosynthetic enzymes, and GA3oxs catalyze the final step in the generation of bioactive GAs (GA1, GA3, and GA4)^36–38^. The expression levels of *GA20ox1*, *GA3ox1*, and *GA3ox2* were dramatically increased in *SlOFP20*-OE plants compared with wild-type plants (Fig. 6d–f).The expression levels of *GA2ox1* and *GA2ox2*, which encode GA2oxs (the main GA catabolic enzymes)^39^, were also detected, and the data suggested that the levels of both *GA2ox1* and *GA2ox2* were sharply increased in *SlOFP20*-OE plants (Fig. 6g, h).

**Fig. 7:**
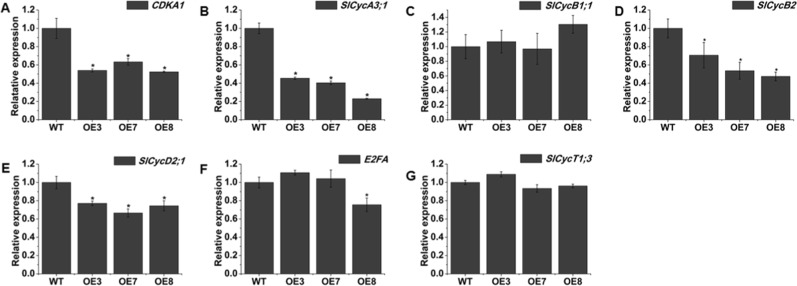
Relative mRNA transcription levels of tomato cell division-related genes in WT and overexpression lines. **a**–**g** represent *CDKA1*, *SlCycA3;1*, *SlCycB1;1*, *SlCycB2*, *SlCycD2;1*, *E2FA*, and *SlCycT1;3*, respectively. Each value represents the mean ± SE of three replicates. * indicate a significant difference (*P* < 0.05) between the wild-type and transgenic lines

**Fig. 8:**
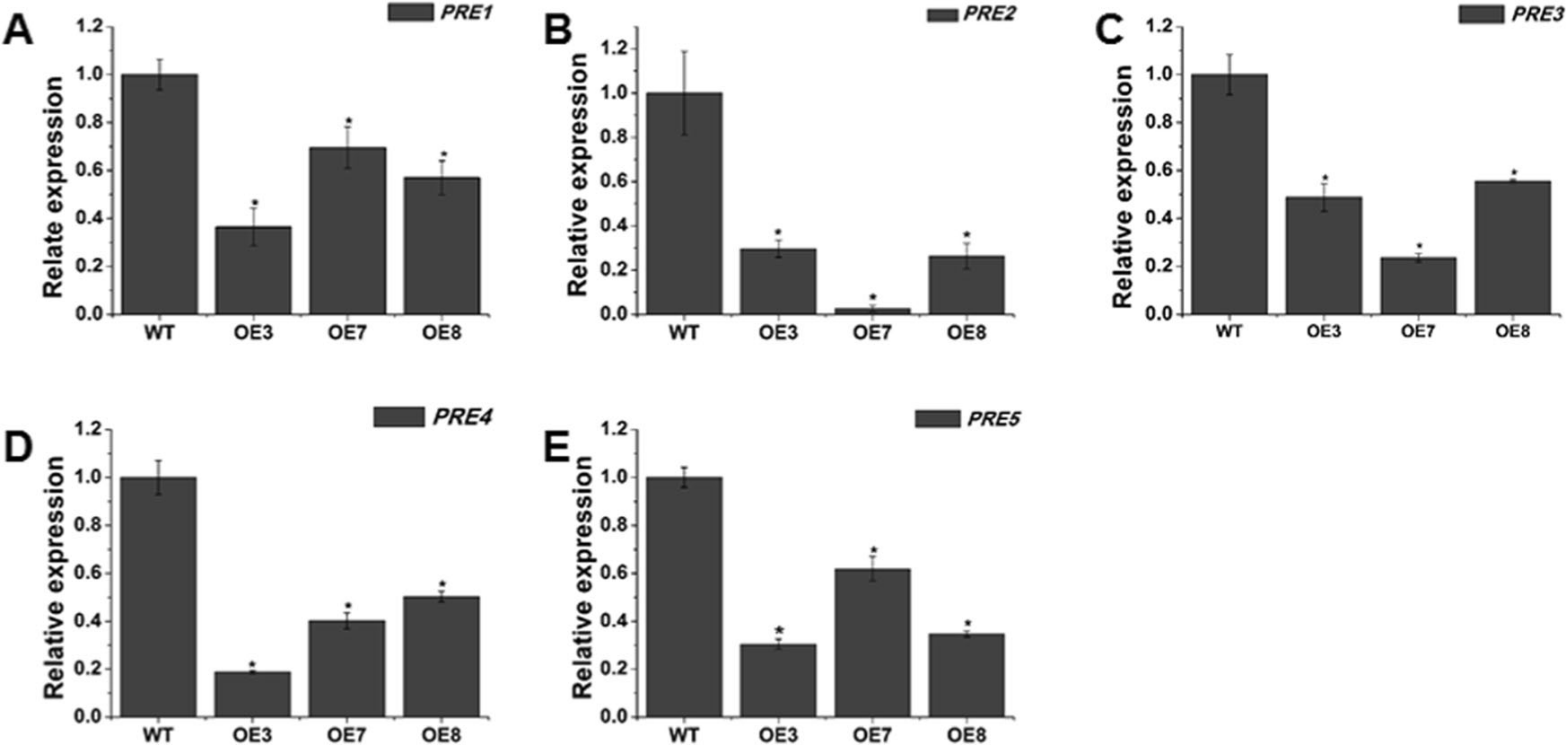
Expression levels of genes involved in cell elongation in WT and transgenic plants. **a**–**e** represent *PRE1*, *PRE2*, *PRE3*, *PRE4*, and *PRE5*, respectively. Each value represents the mean ± SE of three replicates. * indicates a significant difference (*P* < 0.05) between the wild-type and transgenic lines

Generally, the length of plant organs is determined by cell number and cell length, which are associated with cell division and cell elongation, respectively. There are hundreds of target genes downstream of the BR and GA pathways, including cell division and cell elongation genes; thus, we attempted to detect the transcript levels of some genes associated with cell division and cell elongation by RT-qPCR. The cell cycle regulatory gene *CDKA1* was distinctly downregulated in *SlOFP20*-OE transgenic lines (Fig. 7a). The transcription accumulation of four cyclin genes was checked. *SlCycA3;1*, *SlCycB2*, and *SlCycD2;1* were notably repressed in *SlOFP20*-OE transgenic lines (Fig. 7b, d, e), but *SlCycB1;1* (Fig. 7c) was not affected. We also detected the mRNA transcript levels of *E2FA* and *SlCYCT1;3* (Fig. 7f, g), two cell cycle-associated genes that participate in the G1 to S transition, but no distinct changes were found between the WT and *SlOFP20-OE* transgenic lines. The PRE (Paclobutrazol resistance) family of small helix-loop-helix (HLH) proteins positively regulates plant cell elongation^40,41^. There are five putative *PREs* (*PRE1*-*5*) in tomato, and the expression levels of these five genes were examined (Fig. 8a–e); compared to WT, all of them were notably suppressed in the *SlOFP20*-OE transgenic lines.

### Overexpression of *SlOFP20* reduces male fertility

**Fig. 9:**
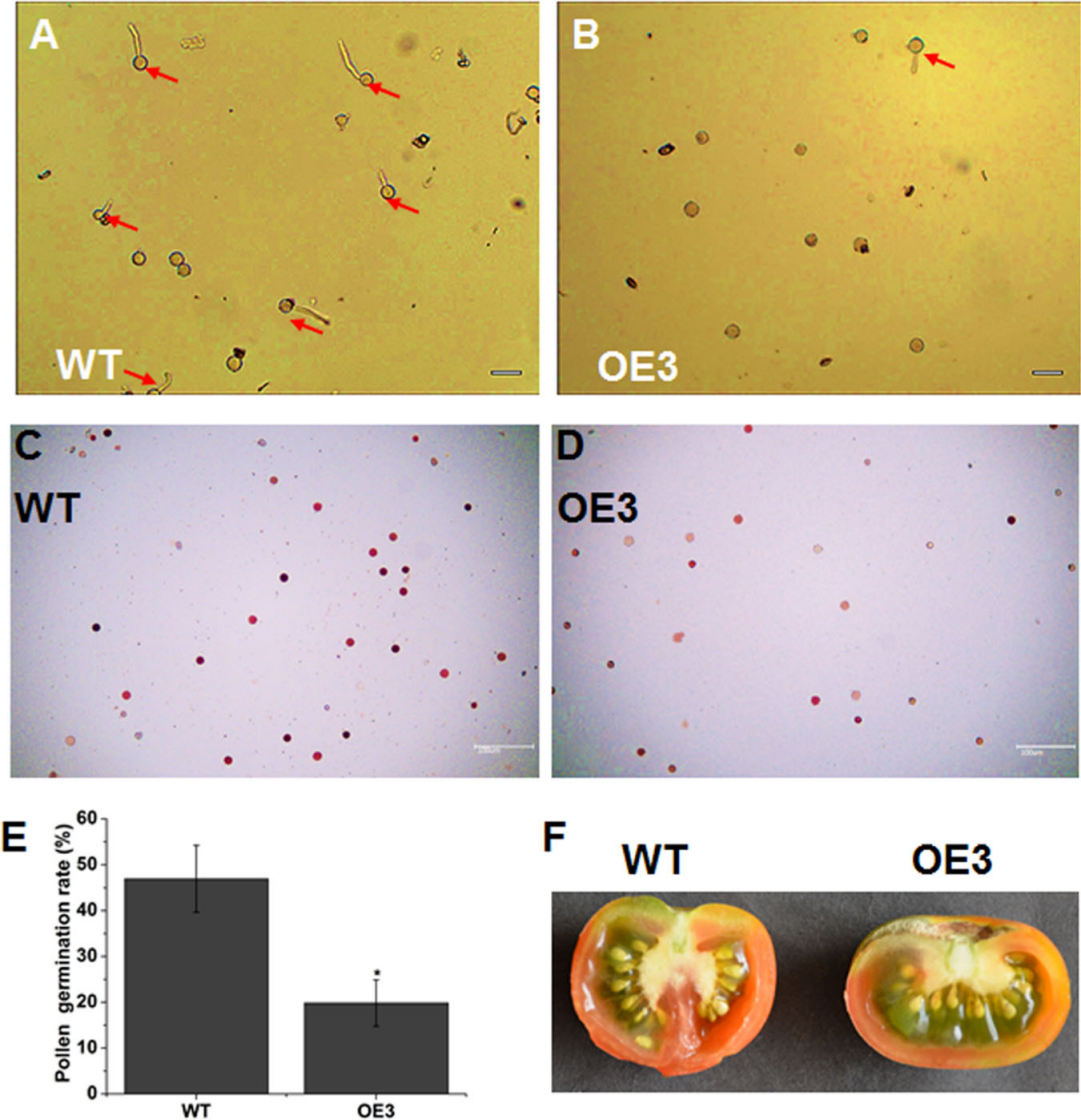
Overexpression of *SlOFP20* reduces male fertility. Pollen germination of WT (**a**) and *SlOFP20*-OE lines (**b**). The red arrow indicates germinated pollen. Bars = 50 μm. Comparison of the pollen germination of WT (**c**) and *SlOFP20*-OE (**d**) with TTC staining. Bars = 100 μm. **e** Comparison of pollen germination rates between WT and *SlOFP20*-OE lines. **f** The fruit of the transgenic line generates normal seeds in a manual crossing assay

In this study, we found that the strong *SlOFP20*-OE tomato plants could not bear fruit, which may be due to the exserted stigmas of *SlOFP20*-OE tomato flowers. We wondered whether the development and function of the pollen in *SlOFP20*-OE tomato flowers was also affected. The quality of pollen is crucially important in the reproductive stage of most plant species. Hence, a pollen germination experiment was carried out to evaluate the impact of *SlOFP20* overexpression on pollen vitality. The results suggested that the pollen germination rate of transgenic plants was distinctly lower than that of WT plants (Fig. 9a, b). Further statistical analysis indicated that the pollen germination rate of *SlOFP20*-OE plants was 2.36-fold lower than that of WT plants (Fig. 9e). Moreover, TTC staining for pollen viability suggested that there were fewer pollen viable grains in *SlOFP20*-OE flowers than in WT flower. These results suggested that overexpression of *SlOFP20* may impair male fertility. When the flowers of *SlOFP20*-OE plants were manually crossed with WT pollen, normal seeds could develop, hinting that the female fertility of *SlOFP20*-OE transgenic plants may not be affected (Fig. 9f).

**Fig. 10:**
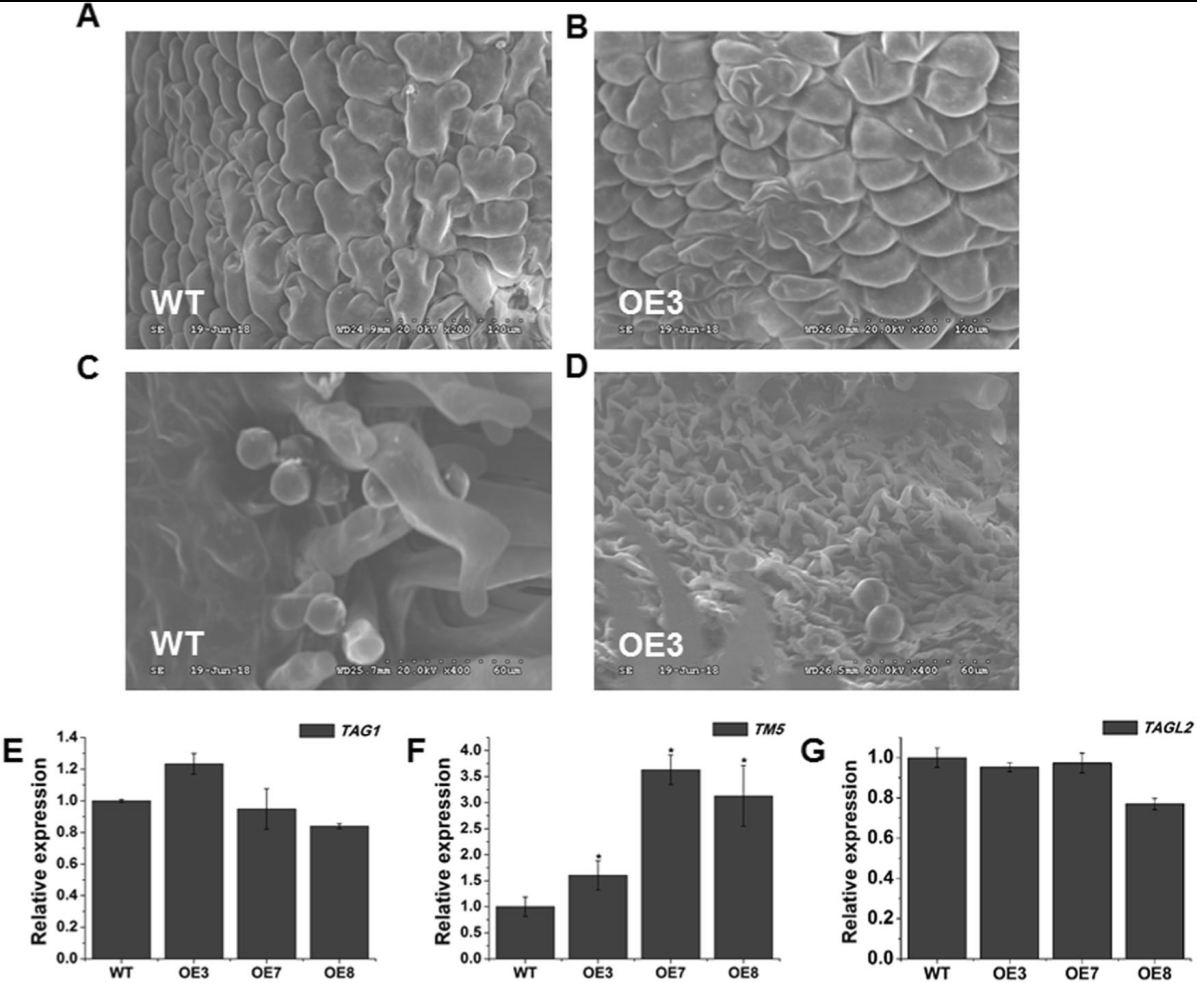
Overexpression of *SlOFP20* affects stamen morphology. Overexpression of *SlOFP20* affects stamen morphology. Electron microscopy observations of epidermal cells of the stamen and pollen in WT (**a**, **c**) and *SlOFP20*-OE transgenic lines (**b**, **d**). **a**, **b** Morphology of epidermal cells of the stamen. **c**, **d** Morphology of pollen grains. The expression levels of floral organ identity genes in WT and *SlOFP20*-OE lines. *TAG1* (C-class gene) (**a**), *TM5* (**b**) and *TAGL2* (E-class genes) (**c**) in WT and transgenic lines. Each value represents the mean ± SE of three replicates. * indicates a significant difference (*P* *<* 0.05) between WT and transgenic lines

A scanning electron microscope was employed to observe the stamen morphologies of WT and transgenic plants. The stamen epidermal cells of the transgenic plants were shorter and more intense than those of WT, and the cell arrangement in OE3 had a scale-like appearance (Fig. 10a, b). The shape of the pollen grains in WT and the OE3 transgenic plants did not show obvious changes (Fig. 10c, d). The grains of both plant lines presented a normal globular shape. In addition, we detected three floral organ identity genes, *TAG1*, *TAGL2*, and *TM5*. *TAG1* is a C-class gene that has been suggested to take part in the specification of stamen and carpel identities^42^. *TM5*^43^ and *TAGL2* (syn. *TM29*)^44^ are E-class genes. The expression levels of *TAG1* and *TAGL2* were not obviously altered in *SlOFP20*-OE transgenic lines (Fig. 10e, g), while that of *TM5* was evidently increased in *SlOFP20*-OE transgenic plants (Fig. 10f).

**Fig. 11:**
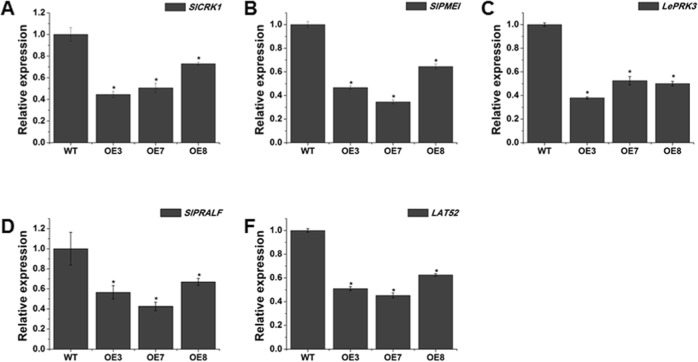
Expression analysis of tomato pollen-specific genes in the pollen of WT and transgenic plants. (**a**–**e**) represent the expression of the pollen-specific genes *SlCRK1*, *SlPMEI*, *LePRK3*, *SlPRALF* and *LAT52* in the pollen of WT and transgenic lines. Each value represents the mean ± SE of three replicates. * indicates a significant difference (*P* < 0.05) between WT and transgenic lines

Moreover, the expression levels of pollen development-specific genes were evaluated in WT and *SlOFP20*-OE tomato plants. *SlCRK1*, a cysteine-rich receptor-like kinase, plays a critical role in pathogen protection and programmed cell death^45^. Pectin methylesterase inhibitor (*SlPMEI*) acts as a key regulator of pectin methylesterase (PME)^46^. *LePRK3*, a pollen-specific receptor kinase gene, may take part in perceiving extracellular cues during pollen tube growth^47^. *SlPRALF*, an exogenous rapid alkalinization factor, negatively adjusts the elongation of the pollen tube^48^, and *LAT52* may participate in germination or early tube growth^49^. The results showed that all five of these genes were remarkably repressed in *SlOFP20*-OE lines, indicating that the overexpression of *SlOFP20* suppressed the mRNA expression of these genes, resulting in reduced pollen fertility (Fig. 11a–e).

Mounting evidence suggests that numerous cis-elements play a vital role in specifying the tissue expression patterns of plant genes^50,51^. To understand what drives the expression of *SlOFP20* in the pollen, a 2000-bp region upstream of the *SlOFP20* start codon was submitted to a public database (http://www.dna.affrc.go.jp/PLACE) to analyze cis-acting elements to determine whether pollen development-associated elements were present in the promoter. There were ten pollen-specific activation-related elements POLLEN1LELAT52^52^ and ten late pollen gene g10-related elements^53^ identified in the *SlOFP20* promoter (Supplementary Table S2). This result further demonstrated that *SlOFP20* may participate in pollen development.

### Mild overexpression of *SlOFP20* in tomato may promote seed germination

**Fig. 12:**
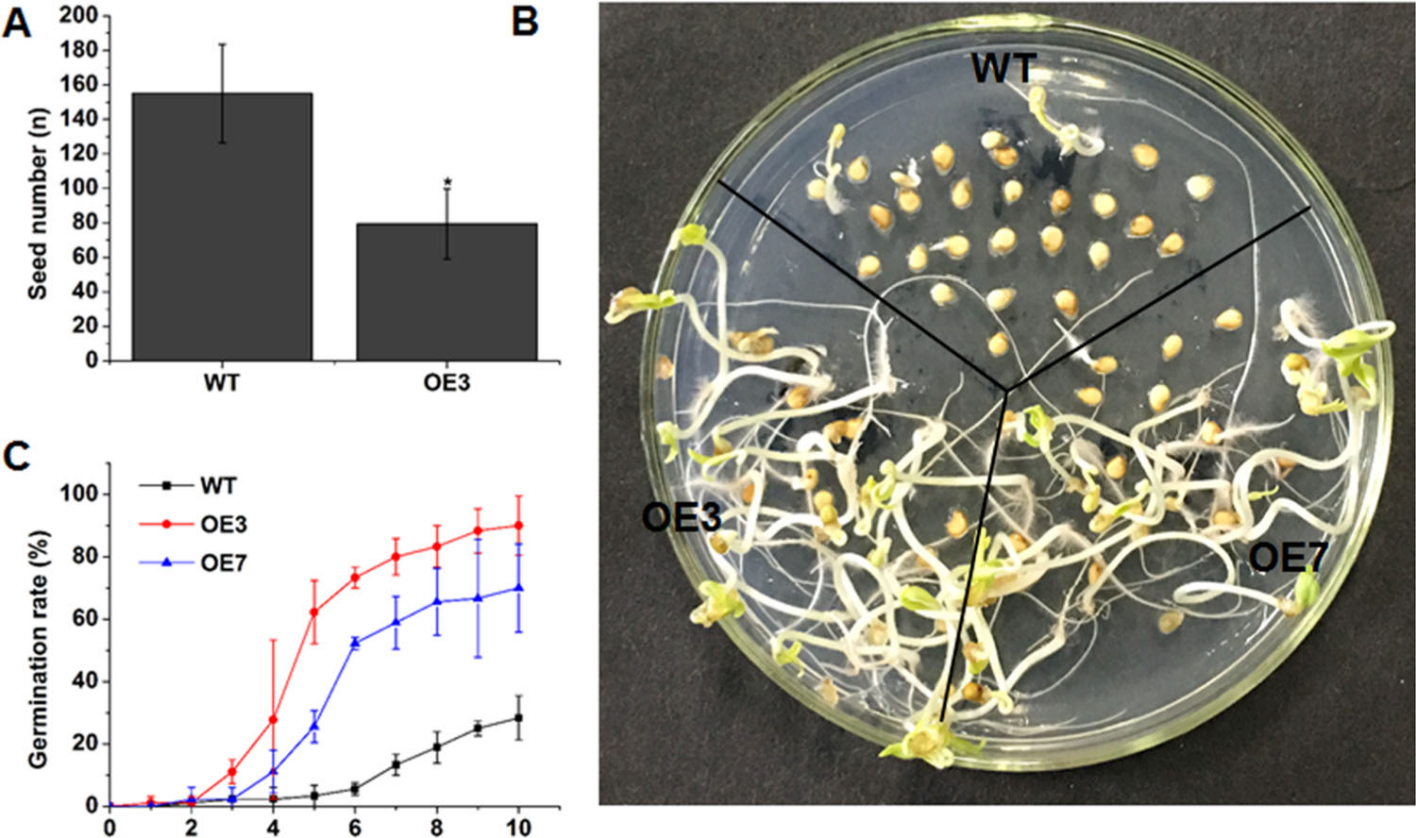
Mild overexpression of *SlOFP20* in tomato may promote seed germination. **a** The seed number per fruit of WT and mild *SlOFP20*-OE transgenic plants. Values are presented as the means ± SEs of measurements from 20 independent fruit. **b** Germination phenotype of WT and *SlOFP20*-OE transgenic seeds. **c** Germination rates of seeds on MS medium. Seed germination was scored every day. The data are the means ± SEs from three replicates with 30 seeds per replicate

In this study, the plants with mild overexpression of *SlOFP20* could generate fruits with seeds. However, compared to WT, the seed number of OE3 transgenic plants was reduced by ~49%, which may be partially due to the reduced male fertility of *SlOFP20*-OE transgenic plants (Fig. 12a). As the seed number was decreased in the transgenic plants, we sought to determine the germination energy of transgenic tomato seeds; thus, a seed germination experiment was performed. The results indicated that *SlOFP20*-OE transgenic seeds exhibited notably higher germination rates than those of WT (Fig. 12b, c), implying that mild overexpression of *SlOFP20* in tomato may speed up transgenic tomato seed germination.

## Discussion

The *OVATE* gene is initially found in tomato and shown to act as a plant-growth suppressor^5^. Extensive studies have since focused on the functional analysis of this family in *Arabidopsis* and rice. As this research has progressed, OVATE family proteins have come to be considered vital regulators involved in organ shape and size determination^5,9^, secondary cell wall formation^10^, embryo sac development^19^, fruit ripening^16,17^, vasculature development^12^, vegetative to reproductive phase transition^11^, male transmission and pollen function^6^, and phytochrome signaling^7,13–15^. However, since the functional description of *OVATE*, little attention has been paid to the other OVATE family proteins in tomato. *SlOFP20*, a member of the OVATE family proteins, was isolated and functionally studied by using overexpression technology. Overexpression of *SlOFP20* in wild-type tomato distinctly impacted the development of the vegetative and reproductive phases. In this paper, we were particularly concerned with the influence of *SlOFP20* on the regulation of reproductive development. The strong overexpression of *SlOFP20* in tomato resulted in exserted stigmas, an altered floral architecture and an absence of fruit, indicating that the overexpression of *SlOFP20* affected the development of reproductive processes.

Overexpression of *OVATE* produces pleiotropic phenotypes in tomato plants, including exserted stigmas, smaller floral organs, and round fruit^5^. In our study, the floral architecture of the strong *SlOFP20* overexpression lines resembled that of *OVATE* overexpression lines. Similar phenotypes related to flowers have been found in *Arabidopsis*^7^ and rice^15^, indicating that OFPs exhibit overlapping functions in controlling plant growth and development. Statistical data suggested that the sepals, petals, and stamens of *SlOFP20*-OE plants were much shorter and wider than those of WT plants. The exserted stigma phenotype of strong *SlOFP20*-OE plants may be ascribed to uneven repression of the growth of different floral organs, similar to what is observed upon the overexpression of *ovate* in tomato.

BRs are a group of polyhydroxylated steroid hormones that play central roles in controlling plant growth and development and conveying various environmental inputs^54,55^. The primary BR signaling pathway has been well established in *Arabidopsis*. Briefly, BRs directly bind to BRI1 (BRASSINOSTEROID-INSENSITIVE1), which belongs to the plasma-membrane-localized and leucine-rich repeat (LRR) receptor kinases^56–58^, thereby activating it and triggering a signal transduction cascade. Finally, the accumulation of unphosphorylated transcription factors BZR1/ BES1 in the nucleus modulates BR-responsive genes by binding target DNA^59,60^. OsOFP1^14^, OsOFP8^13^, and OsOFP19^15^ have been proposed to take part in the regulation of BR signaling. Considering the similar phenotypes observed in *SlOFP20*-OE tomato plants and *OsOFP19*-OE rice plants, we wondered whether the regulatory mechanisms of SlOFP20 and OsOFP19 affecting plant growth and development were conserved in tomato and rice. Ectopic expression of the rice homeobox gene *OSH1* in tobacco generates thicker and shorter leaves^61^, and loss-of-function of DWARF AND LOW-TILLERING (*DLT*) results in a semidwarf phenotype with wider and shorter leaves^62^. OsOFP19 directly interacts with OSH1 to increase the transcriptional activity of OSH1, resulting in a transition of the cell division pattern and antagonizing DLT in BR signaling, which positively regulates BR signaling^15^. Thus, we attempted to examine the transcript levels of the homologous genes of *OSH1* and *DLT* in tomato, *KNOX1* and *GRAS41*. The results suggested that the mRNA accumulation of *KNOX1* in *SlOFP20*-OE transgenic lines was sharply increased. In contrast, the mRNA accumulation of *GRAS41* was notably decreased. We also tried to examine the interactions between SlOFP20, KNOX1, and GRAS41 by using a yeast two-hybrid assay, and positive results were obtained. KONX1 and GRAS41 exhibited a clear interaction, indicating that SlOFP20, KNOX1, and GRAS41 can form a complex. To determine the influence of the overexpression of *SlOFP20* on BR metabolism, we assessed the expression of *CPD*, *D2*, and *DWARF*, which are key genes for BR biosynthesis. The expression of *CPD*, *D2*, and *DWARF* was significantly promoted in *SlOFP20*-OE transgenic plants. In addition, we evaluated the transcript abundance of *CYP734A7*, encoding a key BR catabolic enzyme. The transcript level of *CYP734A7* was also increased in *SlOFP20*-OE transgenic plants. These results are consistent with observations made in *OsOFP19*-OE transgenic plants. In addition, we noted that the upregulation of BR biosynthesis genes may be due to feedback regulation by BR signaling, which was not discussed in relation to *OsOFP19*-OE transgenic plants. *OsOFP19*-OE plants show greatly reduced sensitivity to 24-epibrassinolide treatment, indicating that *OsOFP19* plays a negative role in the BR response^15^. In rice, OSH1 promotes the mRNA accumulation of BR degradation-related genes, and inducible overexpression of *OSH1* results in insensitivity to BR^63^. Previous studies have suggested that BR-insensitive mutants exhibit increased transcript abundance of BR biosynthesis genes and a higher BR content^35,64^. BRI1 is essential for the homeostasis of endogenous BR contents^35^. Therefore, we measured the transcript level of *BRI1*, which was notably increased in *SlOFP20*-OE transgenic plants. Similar characteristics are found in the rice *dlt* mutant, which also shows less sensitivity to BRs. The BR biosynthesis genes *D2*, *D11*, *OsCPD*, and *OsBR6ox* are all upregulated, as is the BR signaling gene *BRI1*^62^. In our study, the *GRAS41* gene homolog of *DLT* exhibited reduced levels in transgenic plants. Whether KNOX1 and GRAS41 participate in BR signaling has not yet been reported in tomato. Based on the above results, we speculate that SlOFP20 may negatively regulate the BR response in tomato, similar to OsOFP19 in rice. In addition, the altered floral architecture of *SlOFP20*-OE plants may mainly be due to the reduced BR response.

The phenotypes of BR-deficient or BR-insensitive mutants are similar to those of GA-deficient or GA-insensitive plants. Many studies have focused on the question of whether BRs may regulate growth by impacting GA biosynthesis. BR treatment and overexpression of *DWF4* increase the expression of three GA biosynthesis genes, *GA20ox1*, *GA20ox2* and *GA20ox5*, in *Arabidopsis*^65^. In addition, BRs have been found to modulate GA biosynthesis in rice^66^. Hence, we assumed that GA metabolism was altered in the *SlOFP20*-OE transgenic lines. To investigate this hypothesis, the expression levels of GA biosynthesis genes, including *CPS*, *KS*, *KAO*, *GA2Oox1*, and *GA3ox1* and *GA3ox2*, were measured. All of these genes showed increases in *SlOFP20*-OE transgenic plants. Moreover, we detected the mRNA abundance of the GA inactivation genes *GA2ox1* and *GA2ox2*. The expression of *GA2o*x1 and *GA2ox2* was increased. Interestingly, the mRNA accumulation of all of the examined BR and GA metabolism genes was found to be increased in our study. Previous work has shown that high levels of BRs induce GA inactivation by increasing the expression of *GA2ox*-*3* to counter the increase in GA biosynthesis due to increased *GA3ox*-*2* expression, finally resulting in growth inhibition^66^. As stated above, BR-insensitive mutants exhibit a higher content of BR, and SlOFP20 may negatively control BR signaling. Thus, we speculate that overexpression of *SlOFP20* in tomato reduces BR signaling, resulting in the accumulation of BR, which then induces the mRNA accumulation of the GA inactivation genes *GA2ox1* and *GA2ox2* to counteract the increase in GA biosynthesis due to increased expression of *CPD*, *KS*, *KAO*, *GA20ox1*, *GA3ox1*, and *GA3ox2*, ultimately leading to growth inhibition.

There are hundreds of target genes downstream of the BR and GA pathways, including cell division and cell elongation; thus, the expression levels of cell division genes (*CDKA1, SlCycA3;1*, *SlCycB1;1, SlCycB2*, *SlCycD2;1*, *E2FA*, and *SlCYCT1;3*) and cell elongation genes (*PRE1*-*5*) were investigated. Four cell division genes, *CDKA1*, *SlCycA3;1*, *SlCycB2*, and *SlCycD2;1*, were significantly suppressed in the *SlOFP20*-OE transgenic plants. Overexpression of *SlPRE2* in tomato promotes the elongation of plant stem internodes^67^. In our study, the downregulation of *SlPREs* illustrated that cell elongation may be impaired in *SlOFP20*-OE transgenic plants. The SEM observations of the stamen surface also supported this result. Therefore, overexpression of *SlOFP20* repressed cell division and cell elongation.

The key yield components of most crop species are fruit and seeds. Therefore, extensive studies have focused on fruit and seed development for decades^68^. In our study, transgenic tomato plants with strong overexpression of *SlOFP20* bore no fruit. BR-deficient and BR-perceptional mutants, including *cpd*, *dwf4*, and *bri1*, are male sterile or show significantly reduced male fertility due to shortening of the stamen and defects in pollen development, and these developmental defects correlate with the inhibition of several critical genes that participate in the development of anthers and pollen, indicating that BRs are critical for plant reproductive development^69–71^. As mentioned above, overexpression of *SlOFP20* reduced the BR response. Here, we also observed reduced length of the stamen, resulting in an exserted stigma phenomenon, which blocked the normal pollination process. On the other hand, a pollen germination experiment was carried out to examine the germination ability between WT plants and *SlOFP20*-OE transgenic tomato plants. The results showed that the pollen germination rate of the transgenic plants was significantly decreased, and this result was further supported by TTC staining, indicating that overexpression of *SlOFP20* inhibits the normal development of pollen grains, but their form was not significantly altered when observed by SEM. In addition, a manual crossing assay was performed to verify that the maternal fertility of *SlOFP20*-OE transgenic lines was not affected. Furthermore, RT-qPCR analysis was conducted to check the transcript accumulation of tomato pollen-associated genes, including *SlCRK1*, *SlPMEI*, *LePRK3*, *SlPRALF*, and *LAT52*. All of these genes showed a trend of downregulation in the transgenic lines, suggesting that *SlOFP20* may control the mRNA accumulation of these pollen-specific genes to impact pollen development. In addition, previous studies revealed that many pollen-specific cis-acting elements, including the pollen-specific activation-related elements POLLEN1LELAT52 and the late pollen gene g10-related elements, were enriched in the promoter regions of two pollen-specific genes, *SlCRK1*^45^ and *SlPMEI*^46^. Promoter-GUS chimeric expression experiments have been used to confirm that the promoters of *SlCRK1*^45^ and *SlPMEI*^46^ exhibit strong pollen-specific activity in the transgenic *Arabidopsis* and tomato plants. In our study, 10 pollen-specific activation-related elements POLLEN1LELAT52 and 10 late pollen gene g10-related elements were found in the *SlOFP20* promoter, which further suggests that *SlOFP20* may play an important role in pollen development. Similarly, *AtOFP1* has been demonstrated to be essential for pollen function^6^.

Moreover, tomato plants with mild overexpression of *SlOFP20* can bear fruit. However, the seed number per fruit in the transgenic plants is sharply reduced compared with that in WT plants, which is in accord with the impairment of the pollen germination rate in transgenic plants. In addition, a seed germination experiment was performed to assess the quality of *SlOFP20*-OE transgenic seeds, and the results clearly showed that the germination rate of transgenic plants was higher than that of WT. AtOFP5 has been demonstrated to interact with BLH1 and KNAT3, which guarantees normal embryo sac development in *Arabidopsis*^19^. Therefore, we assumed that *SlOFP20* may participate in the development of the embryo sac, and more elaborate experiments should be performed to verify this possibility in the future.

In conclusion, the major objectives of this study were to investigate the influence of the overexpression of *SlOFP20* on the reproductive development of tomato. Overexpression of *SlOFP20* in tomato not only altered the morphology of the flowers and fruit but also reduced male fertility. In addition, mild overexpression of *SlOFP20* may accelerate the germination rate of transgenic lines. Analyses of morphological, physiological, and molecular features have been performed to preliminarily elucidate the causes of *SlOFP20*-OE plants defects. A working model is proposed to explain the functions of SlOFP20 in plant growth and development (Fig. S3). Briefly, strong overexpression of *SlOFP20* may directly inhibit the BR response via promoting the expression of *KNOX1* and suppressing the expression of *GRAS41*, leading to changes in a large number of genes, such as genes affecting cell division, cell elongation and pollen development. On the other hand, the reduced BR response in *SlOFP20*-OE plants leads to the accumulation of BR, which then induces the expression of GA inactivation genes to counteract the increase in GA biosynthesis. These results highlight that SlOFP20 functions as an important transcription factor to modulate reproductive development in tomato plants. Thus, it is meaningful to identify the biological function of SlOFP20 or other OVATE family proteins, which will not only extend knowledge of the biological functions of OVATE family proteins but also provide new insight for exploring the importance of OFPs in controlling plant vegetative growth and reproductive development. Considering that OFPs have been suggested to share overlapping functions with other OFP gene family members, a gain-of-function approach was used to study the functions of *SlOFP20* in this study. In future studies, CRISPR/Cas9 genome editing may be a better way to generate *Slofp20* mutants for gene functional studies. To identify the protein partners and targeted genes of SlOFP20, it can be beneficial to elucidate the molecular mechanisms by which SlOFP20 modulates plant growth and development.

## Supplementary information

Supplementary Figures

Supplementary Tables
