# Supplementary material for: Overexpression of SlOFP20 affects floral organ and pollen development
Source: Hortic Res. 2019 Nov 15;6:125. doi: 10.1038/s41438-019-0207-6 (PMC6856366; doi:10.1038/s41438-019-0207-6)
Supplement: Supplementary file 1 — Supplementary Figures [file 41438_2019_207_MOESM1_ESM.docx]

Supplementary Fig. S1


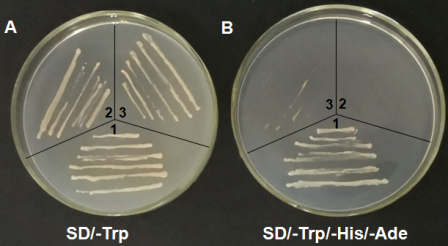


Supplementary Fig. S1. Analysis of the transactivation activity of SlOFP20. SlOFP20 and GAL4 DNA-binding domain fusion protein were expressed in the yeast strain Y2HGold. Vectors pGBKT7 and pGBKT7-53+pGADT7-RecT were expressed in yeast as a negative and positive control, respectively. The yeast streak was cultured on SD/–Trp and SD/–Trp/–His/–Ade medium.

1, pGBKT7-53 and pGADT7-T (positive control); 2, pGBKT7 (negative control); 3, pGBKT7-SlOFP20.

Supplementary Fig.S2


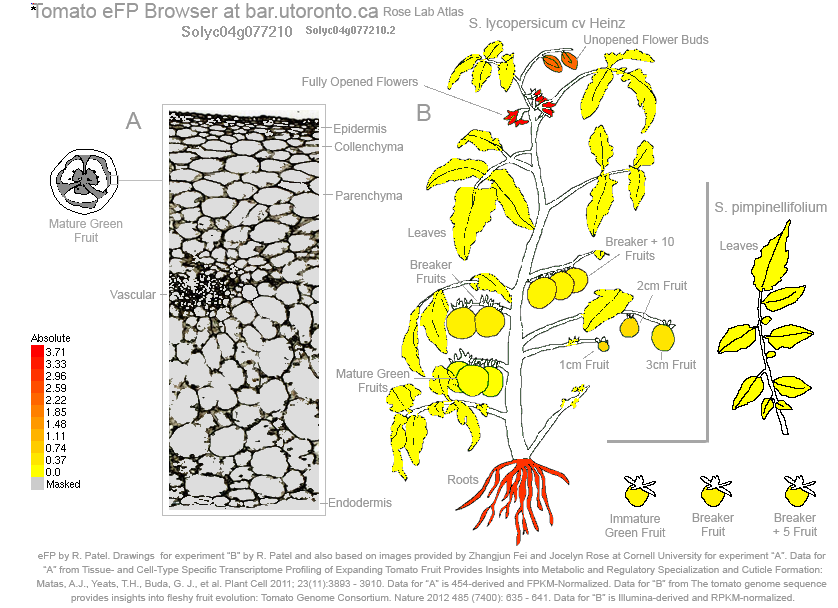


Supplementary Fig.S2. The expression profile of *SlKNOX1* in the Tomato eFP Browser.

Supplementary Fig. S3


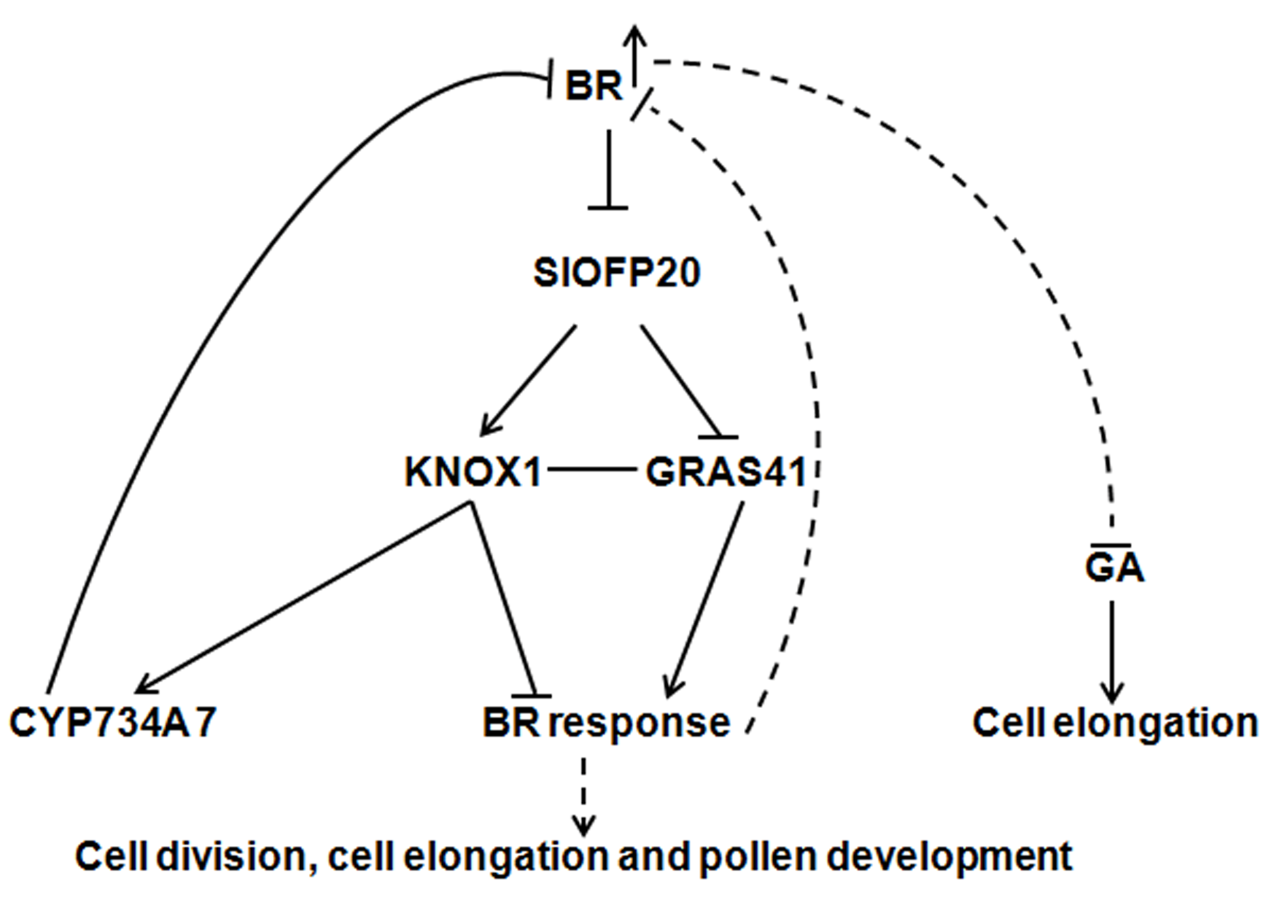


Supplementary Fig.S3. A working model for the cooperation of SlOFP20, KNOX1and GRAS41 in BR response and plant growth and development.
