# Supplementary material for: Overexpression of SlOFP20 affects floral organ and pollen development
Source: Hortic Res. 2019 Nov 15;6:125. doi: 10.1038/s41438-019-0207-6 (PMC6856366; doi:10.1038/s41438-019-0207-6)
Supplement: Supplementary file 2 — Supplementary Tables [file 41438_2019_207_MOESM2_ESM.docx]

Supplementary Table S1. Specific primers used in this study.

| Primer name | Primer Sequence (5' →3') | Application |
| --- | --- | --- |
| *SlOFP20* | GCTCTAGA ACTAACTCCAATATCTCCCACTTTC | Overexpression  vector construton |
|  | CGAGCTC TCACGTAGAACATGAGTGTCTGA |  |
| *NPT II* | CTCAGAAGAACTCGTCAAGAAGG |  |
|  | GACTGGGCACAACAGACAATC | Positive transgenic plants detection |
| q*SlCAC* | CCTCCGTTGTGATGTAACTGG | Internal standard gene for quantitative RT-PCR |
|  | ATTGGTGGAAAGTAACATCATCG |  |
| q*SlOFP20* | AACGTCCACATCACTGAGCC | Gene expression detection |
|  | AGGGGTTGAATCAGGTTTCGTC |  |
| q*SlCRK1* | AAAGGGATTCTTCCTGATGGC |  |
|  | TCTCGGGTCCTTCTATGCTACA |  |
| q*SlPMEI* | AAACTCCTATCATTCCAAAACCC |  |
|  | CAATTGCATCTTCATACACCTCTT |  |
| q *LePRK3* | TGTCTGTCGTGGTGAAGAGGTT |  |
|  | AGCTGAGCATGTGCTGTCCC |  |
| q*SlPRALF* | CTTCCTTCTTCAACGACCCTG |  |
|  | CATCGCCCTGTAACTAATGTGG |  |
| q *LAT52* | TAATGGAGACCACGAGAACGA |  |
|  | GGGAATAAACCCAACTCATCAAG |  |
| q*TAG1* | ATGAACTTGATGCCAGGGAGT |  |
|  | GGGGTTGGTCTTGTCTAGGGTA |  |
| q*TAGL2* | CAGCAGCAACATCCTCAATCTC |  |
|  | CACAGCATCCAACCAGGTATCA |  |
| q*TM5* | CTTTGTGATGCTGAGGTTGCTC |  |
|  | TTTCCAGTGCTTCTCGTGTTG |  |
| q*CDKA1* | GTATGTGCCGTGATTGTCTG |  |
|  | AACCCCTGAATAGAACCAAATG |  |
| q*SlCycA3;1* | CTAAGAAAAGAGCAGCAGAAGCA |  |
|  | GATTCCTTATCTTTTTCAGCAACAG |  |
| q*SlCycD2;1* | CTGCCAAAGCCTCAAGCG |  |
|  | CAGTGGAGCTAGTGTCATTCGC |  |
| Sl*CycB1;1* | GTATCTCGCCCCGTAACAAG |  |
|  | TCTCCTCAGGTTTTGGCTTT |  |
| q*SlCycB2* | TGAGCAGGAGAAATGGAA |  |
|  | CATTGTCAGCGACCTTGT |  |
| q*E2FA* | GGGTATGCAGATGTGGCCAA |  |
|  | GCAGGTGTAAGGGGAGAAGGA |  |
| q*SlCycT1;3* | TGTACTGCGTGTTCCTGCAA |  |
|  | GTTGCGACAATCTGCCAGTG |  |
| q*PRE1* | CGAAAGAACGAAAAGAGAGACATT |  |
|  | GCTAGAGCGACGATTGCGAA |  |
| q*PRE2* | TATGTCTGGGAGAAGGTCAAGGA |  |
|  | CGACGATTACGAATTTCAGGAAG |  |
| q*PRE3* | TTCACACTCTCCATAGCAACACAT |  |
|  | TCCTCACTTATCCTTGATCCTCC |  |
| q*PRE4* | ATCAAATCGCTGATCTTGTTTCC |  |
|  | CACTTAATCCATCCACTTCTCTGTGTA |  |
| q*PRE5* | CCTTCTCTCTTTGTCCATAACTTGTC |  |
|  | CCTGATGATTGTCTCGAACCG |  |
| q*CPS* | AGGTCTTGTTTTGGCTCCCC |  |
|  | CAAGTAGTGATGGATGTCTCTGCC |  |
| q*KS* | AATGGGCTAAAGATATGTCGGTG |  |
|  | CATGAGTGACGCTCAAGGTTACAG |  |
| q*KAO* | TGGACTTACACCAAAGGTAGGAA |  |
|  | AAATACATCACTGGACAAGACGG |  |
| q*GA20ox1* | TGTGGACGATGAATGGCGTT |  |
|  | TACCGCTCTGTGTAGGCAAC |  |
| q*GA3ox1* | ATAGGCACCCACCCTTGTATA |  |
|  | GGATGAAAGTGCCTTGTCAAAAT |  |
| q*GA3ox2* | GTAGACCAAAGGAACCCTCAAAT |  |
|  | GCCGAACAGATGAAAGTGCT |  |
| q*GA2ox1* | ATTAAGATCCAATAACACTTCG |  |
|  | TCTTGATTTCACACTATTTGC |  |
| q*GA2ox2* | ACTGAAAGATGGACACTGGATCTC |  |
|  | CCTTCCGTTAGTCATAACCTGCA |  |
| q*CPD* | TGGACGAGGCTAAGAAGATAACG |  |
|  | GGTGGATGAGAAAATAGGCAAAG |  |
| q*DWARF* | GTGAATGAAGCGAAAGGACTG |  |
|  | TCATGTACTTGTGAGCTGAACCA |  |
| q*D2* | GCTAGGCTGGAGGCTTCAAT |  |
|  | TCCAAACGGGCATTCCTTTCT |  |
| q*CYP734A7* | TGGCAGCAATTAGACGAGCA |  |
|  | GAGGCCAAAAGGCATGAACG |  |
| q*GRAS41* | TAGGCCCAACCCTCCTACTC |  |
|  | CGAAAGCCAGATTCAACGCC |  |
| q*KNOX1* | AGATGTTTCCAAGGACCCAGAAC |  |
|  | CCGAACGGGAGCATTACCA |  |
| q*BRI1* | TGCTGGAGTTTGGAGGGAT |  |
|  | GTGAGATGTTGGCTGAGTGATA |  |
| OFP20-BD | CCGGAATTCATGGGGAATTATAGGTTTAGATTATC | Yeast two-hybrid assay |
|  | AAAACTGCAGTTACTTGAGTCGAATTTCAGTGATT |  |
| KNOX1-BD | CCGGAATTCTCCCCTTATTTAATGGAGAATAAT |  |
|  | AAAACTGCAGTTACTGACCCAAACGAAAAGG |  |
| KNOX1-AD | CCGGAATTCTCCCCTTATTTAATGGAGAATAAT |  |
|  | CCGCTCGAGTTACTGACCCAAACGAAAAGG |  |
| GRAS41-AD | CCGGAATTCATGTTGGCTGGTTGTTCTTCTTCA |  |
|  | CCGCTCGAGTCAACTTGGCTGATAATAAGAGGAT |  |

Supplementary Table S2. Putative cis-elements found in the promoter region (2 Kb) of *SlOFP20* in tomato.

|  | Cis-element name | sequence | Function | site number |
| --- | --- | --- | --- | --- |
| 1 | POLLEN1LELAT52 | AGAAA | pollen-specific cis-acting elements | 10 |
| 2 | GTGANTG10 | GTGA | pollen-specific cis-acting elements | 10 |
